# The Heart–Gut Axis in Heart Failure: The Role of Next-Generation Pharmacological Therapies

**DOI:** 10.3390/ijms27062913

**Published:** 2026-03-23

**Authors:** Elia Nunzio Maria Salerno, Isabella Fumarulo, Claudia Mendicino, Marcello Vaccarella, Barbara Garramone, Francesco Gallo, Gerardo Volzone, Andrea Cammuso, Vincenzo Della Candelora, Franco Scaldaferri, Loris Riccardo Lopetuso, Antonio Gasbarrini, Francesco Burzotta, Nadia Aspromonte

**Affiliations:** 1 Department of Cardiovascular Sciences, Fondazione Policlinico Universitario “A. Gemelli” IRCCS, 00168 Rome, Italy; elianunziomaria.salerno01@icatt.it (E.N.M.S.); isabella.fumarulo@guest.policlinicogemelli.it (I.F.);; 2 Department of Cardiovascular Sciences, Catholic University of the Sacred Heart, 00168 Rome, Italy; 3 IBD Unit-Centro di Malattie dell’Apparato Digerente (CEMAD), UOC Medicina Interna e Gastroenterologia, Fondazione Policlinico Universitario “A. Gemelli” IRCCS, 00168 Rome, Italy; 4 Dipartimento di Scienze Mediche e Chirurgiche, Università Cattolica del Sacro Cuore, 00168 Rome, Italy; 5 CEMAD, Digestive Disease Center, Medicina Interna e Gastroenterologia, Fondazione Policlinico Universitario “A. Gemelli” IRCCS, Università Cattolica del Sacro Cuore, 00168 Rome, Italy; 6 Department of Life Science, Health, and Health Professions, Link Campus University, 00165 Rome, Italy; 7 Dipartimento di Medicina e Chirurgia Traslazionale, Università Cattolica del Sacro Cuore, 00168 Rome, Italy

**Keywords:** heart failure, gut microbiota, gut–heart axis, SGLT2 inhibitors, sacubitril/valsartan

## Abstract

Heart failure (HF) is a systemic syndrome in which cardiac dysfunction is closely linked to multiorgan involvement, including the gastrointestinal tract. Increasing evidence highlights the relevance of the gut–heart axis in HF pathophysiology, whereby intestinal hypoperfusion, congestion, and barrier dysfunction promote gut microbiota dysbiosis, systemic inflammation, and adverse cardiovascular outcomes. In parallel, the advent of novel HF therapies, particularly sodium–glucose cotransporter 2 inhibitors (SGLT2i) and the angiotensin receptor–neprilysin inhibitor sacubitril/valsartan, has markedly improved clinical outcomes across HF phenotypes. Beyond their established cardiovascular benefits, these therapies may exert pleiotropic effects that extend to the intestinal environment and the gut microbiota. Through integrated actions on hemodynamics, neurohormonal activation, metabolic pathways, and inflammatory processes, recent data suggest that novel HF drugs may indirectly influence the gut-microbial composition and function. Conversely, the gut microbiota may modulate drug efficacy and result in interindividual variability in therapeutic responses, suggesting a bidirectional interaction between pharmacological treatment and the gut ecosystem. This narrative review summarizes current evidence of gut microbiota alterations in HF and critically examines emerging data on interactions between the gut microbiota and novel HF therapies, focusing on SGLT2 inhibitors and sacubitril/valsartan. Understanding this crosstalk may support the development of microbiota-informed, personalized therapeutic strategies in heart failure.

## 1. Introduction

Heart failure (HF) is a complex and progressive clinical syndrome that extends beyond impaired cardiac pump function, involving multiple organs and regulatory systems. Despite substantial therapeutic advances, HF remains associated with high morbidity, mortality, and healthcare burdens worldwide [1,2]. Traditionally, HF pathophysiology has been interpreted primarily through the lenses of hemodynamic dysfunction and neurohormonal activation. However, this paradigm has progressively evolved toward a more systemic view, recognizing HF as a multisystem disorder in which peripheral organs actively contribute to disease onset, progression, and outcomes [1,3].

Among extracardiac contributors, the gastrointestinal tract has gained increased attention, leading to the formulation of the gut–heart axis concept [3,4]. In HF, reduced cardiac output and venous congestion result in intestinal hypoperfusion, mucosal edema, and impairments in epithelial barrier integrity [3,5]. These changes promote gut microbiota dysbiosis and increased intestinal permeability, facilitating the translocation of microbial components and metabolites into the systemic circulation. This process sustains chronic low-grade inflammation and metabolic dysregulation, which are recognized drivers of HF progression [4,5].

Gut microbiota-derived metabolites play a central role in mediating these effects, with several metabolites associated with adverse cardiovascular remodeling, endothelial dysfunction, and worse prognoses in HF [4,6]. Conversely, other molecules, such as short-chain fatty acids (SCFAs), exert cardioprotective effects by modulating inflammation, endothelial function, and myocardial metabolism [4,6].

In parallel with these biological insights, the therapeutic landscape of HF has been reshaped by the introduction of novel disease-modifying agents, particularly sodium–glucose cotransporter 2 inhibitors (SGLT2i) and the angiotensin receptor–neprilysin inhibitor sacubitril/valsartan. These therapies have demonstrated robust reductions in mortality and hospitalizations across the spectrum of HF phenotypes, independently of other comorbidities, with effects extending beyond classical cardiovascular mechanisms [7,8]. Emerging experimental and clinical evidence suggests that these pleiotropic actions may also involve the modulation of the gut microbiota and its metabolic output [4,9].

This narrative review aims to summarize current evidence of gut microbiota alterations in HF and to critically examine emerging interactions between the intestinal ecosystem and next-generation HF therapies, with a focus on SGLT2 inhibitors and sacubitril/valsartan.

Understanding this bidirectional crosstalk may provide novel pathophysiological insights and support the development of microbiota-informed, personalized therapeutic strategies in heart failure.

## 2. Methods

We performed a literature search in PubMed and MEDLINE to identify studies published up to 28 February 2026, investigating the relationship between heart failure, the gut microbiota, and emerging pharmacological therapies. As MEDLINE is largely indexed within PubMed, both databases were explored to ensure comprehensive coverage. The following search string was applied: (“heart failure” OR “cardiac failure”) AND (“gut microbiota” OR “intestinal microbiota” OR “gut–heart axis”) AND (“SGLT2 inhibitors” OR “dapagliflozin” OR “empagliflozin” OR “canagliflozin” OR “sacubitril/valsartan” OR “angiotensin receptor–neprilysin inhibitor”). The reference lists of selected articles were also screened to identify additional relevant studies. Eligible studies included English-language preclinical and clinical research articles, randomized controlled trials, observational studies, and relevant reviews. Preclinical and clinical studies were initially screened separately and subsequently integrated into the final analysis. Studies not directly related to the topic, non-English publications, and abstracts without full texts were excluded. Study selection was based on title and abstract screening followed by full-text evaluation, with priority given to studies with clear methodological design, clinical relevance, and mechanistic insight.

## 3. The Gut–Heart Axis

### 3.1. Pathophysiological Basis

In physiological conditions, the heart and the intestine are functionally integrated through a complex network of hemodynamic, metabolic, immune, and neurohormonal mechanisms that collectively preserve systemic homeostasis. Appropriate cardiovascular performance supports intestinal structure and function, while intestinal metabolic and immunological activity contributes to cardiovascular stability. This integrated system enables the body to adapt continuously to metabolic demands, dietary intake, and environmental triggers, ensuring the coordinated regulation of circulatory, immune, and metabolic processes. The gastrointestinal tract is highly dependent on cardiac output, receiving approximately 20–25% of the total blood flow in healthy individuals [1,2,3].

This substantial perfusion reflects the high metabolic activity of the intestinal mucosa, which requires continuous oxygen and nutrient supplies to sustain rapid epithelial turnover and secretory function. Preserved splanchnic blood flow supports mucus production, epithelial renewal, and the maintenance of intercellular junctional complexes. In parallel, the splanchnic circulation acts as a dynamic vascular reservoir capable of modulating systemic vascular resistance through autonomic regulation, allowing the adaptive redistribution of blood volume during physical activity, in postprandial states, or under physiological stress [10,11,12]. In this setting, intestinal integrity and function remain closely linked to cardiovascular performance, highlighting the importance of hemodynamic balance in maintaining gut homeostasis [10].

The preservation of intestinal barrier integrity represents a central determinant of systemic homeostasis. The intestinal barrier, with its different layers, plays an important role in preventing bacterial translocation and endotoxin exposure, thereby maintaining low basal inflammatory tone. By limiting systemic immune activation, intact barrier function contributes to the preservation of endothelial integrity and myocardial homeostasis [10]. In this context, metabolic activity within the intestinal lumen further supports cardiovascular regulation. The commensal gut microbiota ferments nondigestible dietary substrates, particularly complex carbohydrates and fibers, generating short-chain fatty acids (SCFAs), with effects on lipid metabolism, glucose homeostasis, and vascular tone through receptor-mediated signaling pathways, thereby contributing to cardiometabolic balance [13,14,15,16].

Beyond SCFAs, the microbial metabolism of amino acids and bile acids generates additional bioactive compounds with physiological relevance. In fact, tryptophan-derived indole metabolites regulate epithelial differentiation and immune responses through the activation of aryl hydrocarbon receptors, resulting in improved immune tolerance and reduced systemic inflammation [10,17,18].

Similarly, the microbial conversion of primary bile acids into secondary bile acids has effects on lipid handling, glucose metabolism, energy expenditure, and vascular function, further linking intestinal metabolic activity to systemic and cardiovascular physiology [13,19]. Furthermore, immune regulation appears to be essential in maintaining controlled exposure to microbial antigens. In this context, some SCFAs support the differentiation and function of regulatory T cells, thereby preserving endothelial function and chronic inflammatory states, directly linked to different cardiovascular diseases [10,13,20]. Another important role is represented by dietary substrates such as choline and carnitine, whose metabolism is linked to hepatic–cardiac–renal function [21,22].

Finally, neurohumoral mechanisms integrate intestinal and cardiovascular function through autonomic and endocrine signaling. The enteric nervous system interacts with central autonomic circuits via vagal and sympathetic pathways, allowing the modulation of gut motility, secretion, and blood flow in response to systemic demands. Entero-endocrine hormones and gut-derived neuroactive mediators further influence vascular tone, cardiac performance, and inflammatory responses [10,21]. Through these mechanisms, the gut acts not only as a digestive organ but also as an active contributor to cardiovascular regulation and systemic homeostasis. Disruption of these tightly regulated physiological mechanisms provides the basis for maladaptive alterations of the gut–heart axis, as observed in heart failure, setting the stage for intestinal dysbiosis, barrier dysfunction, and systemic inflammation.

**Figure 1:**
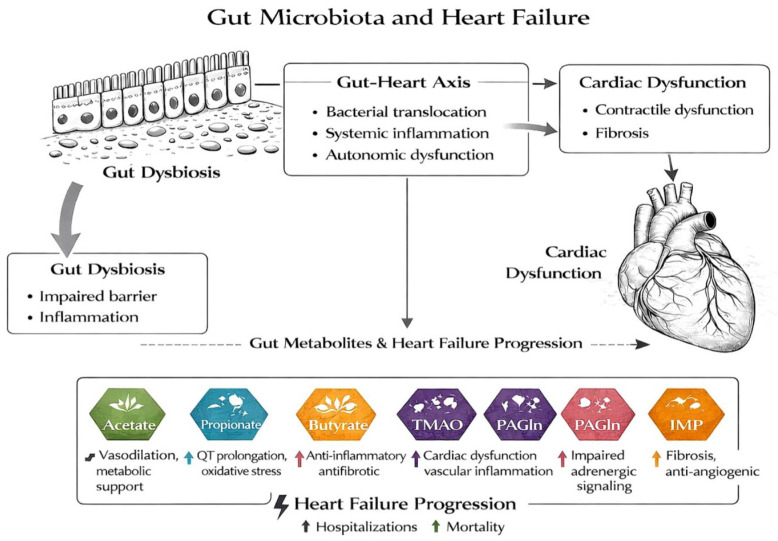
Gut microbiota and heart failure, focusing on the different roles of gut metabolites.

A schematic representation of the relationship between the heart and gut and the mechanisms leading to heart failure is illustrated in Figure 1.

### 3.2. Gut Microbiota Dysbiosis and Cardiovascular Effects

Dysbiosis of the gut microbiota, coupled with impairments in the intestinal barrier, has emerged as a major contributor to systemic inflammation, metabolic dysregulation, and adverse cardiovascular outcomes in heart failure (HF).

In this setting, the gut–heart axis represents a critical interface where microbial alterations translate into clinically relevant cardiac consequences.

Firstly, patients with chronic HF exhibit characteristic shifts in gut microbiota composition, including a reduction in Firmicutes and Bacteroidetes and the expansion of Proteobacteria and Actinobacteria. These changes are consistently associated with compromised intestinal barrier integrity, increased systemic inflammation, and metabolic disturbances that collectively promote HF progression and amplify myocardial damage [23,24,25,26].

Among the downstream mediators of these alterations, gut microbiota-derived metabolites play a central role in HF pathophysiology, with pleiotropic cardiovascular effects, including either accelerating or attenuating disease progression, and with a clinically relevant prognostic and risk stratification impact.

An important metabolite is represented by trimethylamine N-oxide (TMAO), derived by the hepatic conversion of dietary choline and carnitine.

In the myocardium, TMAO promotes hypertrophic growth and fibrotic remodeling while impairing cardiomyocyte contractile performance and mitochondrial function, ultimately leading to reduced energetic efficiency and ATP depletion. These effects translate into the progressive deterioration of systolic function [23,27,28].

At the vascular level, TMAO induces endothelial dysfunction and vascular inflammation, primarily through the activation of pro-inflammatory pathways and oxidative stress, thereby contributing to increased vascular stiffness and impaired vasodilatory capacity. Elevated circulating TMAO levels have been consistently linked to adverse cardiovascular remodeling and HF progression [21,29].

At the cellular level, TMAO disrupts T-tubule organization and calcium handling, leading to impaired excitation–contraction coupling, slower contraction–relaxation kinetics, and reduced mechanical efficiency [23,30].

Furthermore, TMAO reduces nitric oxide bioavailability, amplifying reactive oxygen species accumulation and further compromising myocardial and vascular function, thus reinforcing energy depletion in failing cardiomyocytes and exacerbating mitochondrial dysfunction [23,31].

Beyond direct metabolic and oxidative effects, TMAO also alters neurohumoral regulation, a hallmark of HF. Experimental and clinical data indicate that TMAO enhances sympathetic nervous system activity through both peripheral and central mechanisms, promoting autonomic imbalance and electrophysiological instability, with alterations in microglial P2Y12 receptor signaling [23,32,33].

HF-associated gut dysbiosis also affects phenylalanine metabolism, favoring the increased microbial production of phenylacetic acid (PAA). Elevated levels of this metabolite are strictly linked to metabolic imbalance, cytotoxic effects, and increased short-term mortality risks in HF patients [23,34].

At the myocardial level, an impairment in cardiac performance is observed through the attenuation of adrenergic support for contractility, with a reduction in inotropic reserves and increased cardiac stress markers such as brain natriuretic peptide (BNP), thereby reinforcing maladaptive cardiac remodeling [35,36].

Gut microbiota-derived lipopolysaccharide (LPS) enters the systemic circulation in heart failure due to intestinal barrier disruption. This endotoxemia triggers chronic systemic inflammation and directly aggravates myocardial injury. Circulating LPS levels are inversely associated with cardiac function, with HF-related gut dysbiosis enhancing LPS overproduction [23,37].

Patients with HF display reduced circulating levels of SCFAs, leading to the attenuation of their cardioprotective actions. More specifically, acetate influences cardiac function through direct myocardial and metabolic effects, while propionate improves energy efficiency via glucose utilization but may induce oxidative stress and electrical disturbances, including QT prolongation. Butyrate, in contrast, supports cardiac performance independently of β-adrenergic pathways and exerts anti-inflammatory, antifibrotic, and epigenetic protective actions [23].

Lastly, imidazole propionic acid (IMP), a gut microbiota-derived histidine metabolite, is elevated in heart failure and strongly associated with left ventricular dysfunction, higher NT-proBNP, and increased 5-year mortality, supporting its role as an independent prognostic biomarker. IMP contributes to maladaptive myocardial remodeling by reprogramming energy metabolism and accelerating fibrosis, while also correlating with systemic inflammation and impaired intestinal barrier function. Additionally, IMP exerts anti-angiogenic effects, further impairing myocardial perfusion and promoting HF progression [38,39,40].

A summary of the gut microbiota alterations and all derived metabolites involved in heart failure is provided in Table 1.

## 4. Interactions Between Novel Heart Failure Therapies and the Gut Microbiota

### 4.1. SGLT2 Inhibitors

Sodium–glucose cotransporter 2 inhibitors were initially developed for the treatment of type 2 diabetes mellitus. Their primary mechanism of action involves the inhibition of renal tubular glucose reabsorption, resulting in increased urinary glucose excretion.

However, several large randomized controlled trials have consistently demonstrated the significant clinical benefits of SGLT2 inhibitors in patients with heart failure, regardless of the presence of diabetes and across the entire spectrum of left ventricular ejection fractions. Based on this robust evidence, SGLT2 inhibitors are currently regarded as a cornerstone therapy in contemporary heart failure management.

Despite the well-established clinical efficacy of these agents, the biological mechanisms underlying their beneficial effects in heart failure remain incompletely understood. The observed improvements in heart failure outcomes suggest a broad pleiotropic profile extending well beyond their glucose-lowering properties. Several mechanisms have been proposed, including effects on myocardial metabolism, ion transporters, myocardial fibrosis, adipokine signaling, and vascular function.

In this context, there is growing interest in the potential effects of SGLT2 regarding alterations in the gut microbiota, leading to the publication of several studies—first on mouse models, both diabetic and with heart failure, and then on human subjects.

#### 4.1.1. Murine Studies

The effects of dapagliflozin on the gut microbiota have been studied in a recent work whereby twenty-four diabetic rats without cardiovascular disease were randomized to three groups receiving intragastric infusions of normal saline, metformin, or dapagliflozin for four weeks. After this period, blood glucose levels and plasma insulin levels were determined, and fecal samples were collected to access the microbiome. In addition to a significant reduction in glucose levels, the dapagliflozin group showed higher levels of Ruminococcaceae compared to the group treated with metformin, where high prevalences of Lactobacillaceae and Bifidobacteriaceae were detected. Interestingly, the study revealed that Proteobacteria such as Desulfovibrionaceae, often linked to obesity and diabetes, were enriched in the dapagliflozin group. Given that dapagliflozin was not effective in this study in terms of beneficial bacteria (such as Lactobacillaceae and Bifidobacteriaceae), this research shows that a combination of these two molecules (dapagliflozin and metformin) may be useful in modulating and improving the structure of the fecal microbiota [41].

Additional insights are derived from a recent analysis conducted on the effects of luseogliflozin for eight weeks on sarcopenic obesity, considered a central pathological factor in diabetes. In this research, diabetic rats were randomized to receive luseogliflozin or a low-carbohydrate diet. Skeletal muscle mass, grip strength, and amino acid levels increased in the SGLT2 group compared to the control group. In addition, fecal analysis revealed that, in the luseogliflozin group, there was a significant increase in *Syntrophothermus lipocalidus*, the Syntrophomonadaceae family, *Parabacteroidesdistasonis distasonis*, and the genus *Anaerotignum*, suggesting the role of this molecule in preventing sarcopenic obesity by improving amino acid metabolism, as seen in the group treated with luseogliflozin, thereby being linked to the prevention of sarcopenic obesity [42].

The effects of SGLT2 on cardiovascular disease (CVD) were studied in another work wherein diabetic mice with associated CVD received a high-fat diet for 24 weeks, followed by the use of metformin or canagliflozin for 6 weeks. The results demonstrated that canagliflozin was associated with reductions in serum lipid accumulation and circulating markers of inflammation, as well as improving cardiac mitochondrial homeostasis and relieving oxidative stress. Moreover, canagliflozin improved the ratio of the gut bacteria Firmicutes/Bacteroidetes, with the subsequent restoration of the microbiota composition to close-to-normal levels. These findings suggest additional beneficial effects of this molecule on blood lipids, inflammation, and oxidative stress [43].

An important recent study focused on the effects of SGLT2i in a model of heart failure without diabetes. In this murine model, dapagliflozin treatment was shown to improve endothelial dysfunction and reduce vascular oxidative stress and inflammatory activation. These vascular effects were accompanied by the partial reversal of heart failure-associated gut microbiota dysbiosis, characterized by the selective remodeling of the microbial composition (Firmicutes/Bacteroides ratio). Notably, improvements in endothelial function and microbiota composition occurred in parallel, indicating that vascular function and intestinal conditions may be closely related [44].

Recently, an interesting work was conducted on 39 rats with chronic myocardial infarction, previous significant stenosis on the left anterior descending artery, and heart failure with a left ventricular ejection fraction < 50%. They were randomized to receive dapagliflozin or a placebo for 10 weeks. This model revealed a significant improvement in cardiac function in the dapagliflozin group and the restoration of microbial diversity, with the enrichment of beneficial bacteria (*UCG-007*, Bacillus) and a reduction in harmful taxa (Holdemania). In addition, the authors observed an increase in butyrate production with low plasma TMAO levels, suggesting a favorable microbial metabolic shift [45].

#### 4.1.2. Human Studies

These preclinical and murine observations provide a biological rationale for the exploration of whether similar effects can also be detected in both diabetic and non-diabetic patients with heart failure treated with SGLT2 inhibitors.

A recent review explored the relationship between diabetes, SGLT2i, and the gut microbiota. Specifically, in diabetic patients, significant changes in the gut microbiota were observed, with an increase in harmful bacteria (Firmicutes) and a decrease in beneficial ones (Bacteroidetes). These new drugs work by rebalancing the body’s system through the remodulation of beneficial bacterial and an increase in SCFAs, which are essential in reducing diabetic complications, such as kidney damage, heart problems, and liver fibrosis [46].

In a recent Chinese work, 21 human patients with a new diagnosis of type 2 diabetes mellitus (T2D) undergoing therapy with only canagliflozin were matched with 10 healthy controls. Using RNA sequencing, changes in the gut, oral, and ocular surface microbiotas were observed before and after treatment with canagliflozin. Regarding the gut microbiome, treatment with canagliflozin was associated with an increase in beneficial bacteria such as Lachnospiraceae *UCG 004*, Bacteroides, and Lachnospiraceae *NK4A136*, with a similar composition in healthy controls. Similar changes were identified also in both the oral and ocular surface microbiotas, suggesting a potential role in the global beneficial effects of this molecule [47].

In contrast with this study, another trial investigated the effects of dapagliflozin on the gut microbiota in patients with T2D. In this double-bind randomized trial, 44 diabetic patients were randomized to receive dapagliflozin or gliclazide for 12 weeks. While different effects between the two groups were found on glycemic and metabolic metabolism, neither treatment significantly affected the gut microbiota composition, with the authors concluding that the observed metabolic changes were not mediated by any effects on the microbiota [48].

The impact of empagliflozin on the gut microbiota has been investigated in a recent trial wherein 47 patients with T2D and hypothyroidism were divided into two groups, receiving metformin alone or metformin plus empagliflozin for 6 months. The study found that the combination therapy group had more significant reductions in glucose, glycated hemoglobin, and the atherogenicity coefficient, as well as a lower level of Firmicutes and an increase in the number of Actinobacteria and a higher ratio of Bacteroides fragilis to *Faecalibacterium prausnitzii* [49].

The effects on CVD were studied in a randomized, open-label trial wherein 76 treatment-naïve patients with T2D and CVD risk factors were randomized to receive empagliflozin or metformin for 3 months. Whereas similar results were found for glucose metabolism, only the empagliflozin group showed improvements in CVD risk factors. In addition, significant reshaping in the gut microbiota was identified after 1 month in the empagliflozin group compared to metformin, with an increase in SCFA-producing bacteria such as Roseburia, Eubacterium, and Faecalibacterium and a consequent reduction in several harmful species, including Escherichia–Shigella, Bilophila, and Hungatella [50].

Recently, another interesting study was conducted on 135 patients with heart failure and without T2DM [51]. They were randomized to be treated with dapagliflozin versus conventional therapy. The results showed increased levels of Prevotella, Akkermansia, Collinsella, and Fusobacterium and reduced levels of Bacteroides, Parabacteroides, Subdoligranulum, and Bifidobacterium in the dapagliflozin group. Specifically, the probiotic Akkermansia seems to be involved in enhancing intestinal barrier integrity by degrading the mucus layer and producing anti-inflammatory responses [52]. On the other hand, an analysis of the control group revealed high levels of species such as Lachnoclostridium and the *Ruminococcus gauvreauii* group, often associated with amino acid fermentation and a pro-inflammatory environment [53].

To overcome the limitations of recent observational and cross-sectional studies, the EMPAGUM trials have been specifically designed to prospectively evaluate the effects of SGLT2 inhibition on the gut microbiota in heart failure. This randomized, open-label study is investigating the use of empagliflozin in patients with heart failure with a preserved ejection fraction, incorporating a longitudinal assessment of the gut microbiota composition and diversity and related metabolic and inflammatory profiles. By enabling the longitudinal characterization of gut microbiota dynamics, EMPAGUM is expected to clarify the temporal relationship between SGLT2 inhibition and intestinal microbial changes in heart failure [54].

### 4.2. Sacubitril/Valsartan (ARNI)

Sacubitril/valsartan, a first-in-class angiotensin receptor–neprilysin inhibitor (ARNI), has revolutionized the management of heart failure with reduced ejection fraction (HFrEF). By simultaneously inhibiting the neprilysin enzyme and blocking the angiotensin II type-1 (AT1) receptor, it enhances the beneficial effects of natriuretic peptides while suppressing the deleterious actions of the renin–angiotensin–aldosterone system (RAAS). Beyond these neurohormonal and hemodynamic benefits, emerging evidence suggests that the therapeutic efficacy of sacubitril/valsartan may be partially mediated through its interaction with the gut microbiota.

The gut microbiota is now recognized as a virtual endocrine organ, playing a central role in cardiovascular and metabolic homeostasis. It achieves this through immune modulation, the maintenance of intestinal barrier integrity, and the production of bioactive metabolites. Pathological alterations in microbial composition, called dysbiosis, have been implicated in the progression of heart failure, chronic kidney disease (CKD), and diabetes [4,55].

The emerging field of pharmaco-microbiomics has highlighted that cardiovascular drugs can reshape gut-microbial communities. These drug-induced shifts can contribute to therapeutic efficacy or drive adverse effects [56,57]. While the clinical benefits of ARNI therapy are well documented, its specific interactions with the gut microbiome represent a critical focus in cardiovascular pharmacology.

#### 4.2.1. Murine Studies with ARNI

The only evidence of the efficacy of ARNI in the modulation of the gut microbiota comes exclusively from murine studies. In the first study conducted on rats with diabetic kidney disease, sacubitril/valsartan not only improved renal function and reduced tubulo-interstitial fibrosis but also induced significant changes in the gut microbiota [58]. The treated mice exhibited an increased abundance of potentially beneficial taxa, such as Lactobacillus and Parabacteroides, in addition to a marked reduction in pro-inflammatory microbial signatures, suggesting a possible protective microbial profile that can also mitigate systemic inflammation.

The interaction of ARNI and the gut microbiota was also studied in rat models of chronic intermittent hypoxia, where sacubitril/valsartan partially reversed hypoxia-induced dysbiosis. This shift was accompanied by attenuated aortic inflammation and vascular remodeling [59]. The restoration of microbial diversity and the enrichment of taxa associated with metabolic homeostasis reinforce the possibility of a direct link between this drug, microbiota modulation, and vascular protection.

#### 4.2.2. Murine and Human Studies with RAAS Inhibitors

Although there are few data in the literature on the interactions between ARNI and the gut microbiota—and these data come mainly from animal studies—there is evidence regarding the action of RAAS inhibitors, based on both animal and human studies. In fact, in addition to the well-known effects of these drugs on cardiovascular diseases, it is now also clear that components of the RAAS are expressed locally within the gut. Angiotensin signaling influences microbial ecology and mucosal immune responses. Therefore, AT1 receptor blockade, combined with enhanced natriuretic peptide signaling, may indirectly stabilize the gut environment by improving intestinal perfusion and reducing local oxidative stress [60].

In a murine study with spontaneously hypertensive rats treated with valsartan, it was observed that this molecule decreased the production of the SCFAs isobutyric acid and isovaleric acid, preventing the destruction of the intestinal microbiota and protecting the intestinal mucosal barrier [61].

Another important study, which focused on hypertension, analyzed the effects of irbesartan on the gut microbiota. The results from this study revealed that rats treated with irbesartan experienced the reversal of gut dysbiosis, with a significant increase in *Lactobacillus johnsonii* and *Lactobacillus reuteri*, improved antioxidative and anti-inflammatory abilities, and restored intestinal integrity [62].

The effects of RAAS inhibitors in the modulation of the gut microbiota have also been studied in human models. In a recent work, 36 patients with essential hypertension treated with angiotensin-converting enzyme inhibitors or angiotensin receptor blockers (ACEI/ARB) were compared to 19 hypertensive patients without treatment. The study showed that ACEI/ARB therapy was linked to a reduction in pathological bacteria such as Enterobacter and Klebsiella and increasing levels of beneficial species such as Odoribacter, with associated changes in the production of their respective metabolites [63].

While there are no specific studies on the effects of RAAS inhibitors on the gut microbiota in patients with heart failure, the data obtained regarding the potential effects of these drugs on intestinal dysbiosis in the context of hypertension are encouraging, suggesting that they may also be effective in other cardiovascular settings, such as heart failure.

The interactions between novel heart failure therapies and the gut microbiota are summarized in Table 2.

## 5. Clinical Implications of Drug–Microbiota Interactions

Although there is some emerging evidence that suggests an active relationship between these drugs and the gut microbiota, it is still unclear whether microbiota dysfunction can contribute to the variability in the therapeutic response observed in patients with heart failure.

For example, a recent review highlighted that the metabolism of the gut microbiota can increase the hydrophilicity of certain molecules, potentially increasing their absorption along the gastrointestinal tract [64]. In fact, microbial activity can result in altered drug pharmacokinetics, the activation of prodrugs, the unwanted formation of toxic metabolites, or the inactivation of drugs [65].

The bidirectional association between the gut microbiota and certain drugs was investigated in a murine model based on hypertensive rats and endogenous microbiota dysbiosis treated with losartan. The results from this study revealed a significant reduction in the oral bioavailability of losartan in rats with gut microbiota dysbiosis, suggesting that alterations in the microbiota can contribute to the pharmacokinetic variability of this drug [66].

Nonetheless, these data are derived exclusively from preclinical and murine studies; however, if confirmed in human trials, these findings may partly explain why some patients show a suboptimal response despite optimized pharmacological therapy.

Considering the possible bidirectional interaction between the gut microbiota, drugs, and cardiovascular disease, another interesting aspect is the possibility to modulate the gut microbiota through the diet.

For instance, a prospective clinical dietary intervention study enrolled 100 patients with chronic heart failure. Fifty of these patients were assigned to a dietary regimen characterized by high daily fiber intake, while the remaining 50 maintained a diet with low fiber content. The results showed that patients with high fiber intake had significantly lower levels of circulating TMAO and inflammatory markers, suggesting that dietary fiber could positively regulate the composition of the gut microbiota and reduce dysbiosis-related systemic inflammation [67].

These findings suggest that the combination of medication and targeted dietary regimens (even adding specific probiotics) may represent a potential therapeutic approach for patients with heart failure, but more robust trials are needed to confirm the efficacy of this strategy.

## 6. Future Directions

While the association between HF and microbiota alterations is well established, recent evidence suggests a potential link between new heart failure drugs and their modulation of the gut microbiota. Although most data are derived from preclinical studies or confined to animal models, there is a growing consensus that these factors may be interconnected. Possible therapeutic implications may involve enhancing the effectiveness of therapy through a personalized approach tailored to the patient.

Nevertheless, future well-designed clinical studies in humans, integrating metagenomics, metabolomics, and immune profiling, are essential to clarify the clinical relevance of new heart failure drugs such as ARNI and microbiota interactions and to determine whether the gut microbiome could be a target for the optimization of heart failure therapy.

In this regard, there is growing interest in identifying potential gut microbiota-related biomarkers. In addition to other classic HF biomarkers—such as natriuretic peptides—a recent review suggests the role of SCFAs or TMAO as possible indicators of intestinal dysbiosis, with the significant production of pro-inflammatory cytokines including *IL-1b* and *TNFa* [68].

Further research could therefore clarify whether these biomarkers are useful in the prognostic stratification of patients with heart failure and whether they could inform a personalized therapeutic approach in this population.

At the same time, another problem concerns those patients who do not benefit and who are non-responders to optimized medical therapy. In this area, goals could include the identification of new biomarkers for these patients and the creation of new therapeutic algorithms for these specific conditions.

Fecal microbiota transplantation is a procedure consisting of transplanting stool from a healthy donor into another patient’s intestine. However, there is a lack of evidence of its suitability regarding HF; it has primarily been used in the management of inflammatory bowel disease or recurrent infections, such as those associated with *Clostridium difficile* [69,70]. Pending clinical trials and dedicated studies, FMT may hold promise as a supplementary treatment for HF [71].

## 7. Strength of Evidence Across Therapeutic Classes

For SGLT2 inhibitors, evidence is supported by both preclinical and human studies. Multiple animal models consistently demonstrate that these agents can modulate the gut microbiota composition, improve microbial diversity, and influence metabolite production (e.g., SCFAs and TMAO). In humans, the available data are derived from small, randomized trials and observational studies, mainly conducted in patients with type 2 diabetes and, more recently, heart failure. These studies suggest potential microbiota remodeling and metabolic effects; however, the findings remain heterogeneous and sometimes conflicting. Therefore, while supported by preliminary human data, the clinical relevance and reproducibility of these findings remain to be definitively established.

In contrast, for sacubitril/valsartan, current evidence is limited exclusively to animal studies. Preclinical models indicate that ARNI therapy may influence the gut-microbial composition, reduce pro-inflammatory taxa, and improve intestinal and vascular parameters. However, no studies to date have directly evaluated these effects in human heart failure populations. As such, all microbiota-related mechanisms proposed for ARNI should be considered preclinical and hypothesis-generating.

A relatively stronger level of evidence is available for RAAS inhibitors, although not specifically in heart failure populations. Both animal studies and human investigations in patients with hypertension indicate that ACEI and ARB can modulate the gut microbiota composition, reduce pathogenic bacterial taxa, and increase beneficial species. These findings suggest a potential class effect on the intestinal environment; however, their direct relevance to heart failure remains inferential and has not been specifically confirmed in dedicated HF cohorts.

Beyond these drug-specific observations, several broader mechanisms—such as the modulation of intestinal barrier integrity, systemic inflammation, and host–microbiota metabolic signaling—are supported mainly by indirect evidence or derived from experimental models and therefore remain largely hypothetical in the context of human heart failure.

## 8. Conclusions

Heart failure is increasingly recognized as a systemic syndrome in which cardiac dysfunction is tightly interconnected with alterations in peripheral organs, particularly the gastrointestinal tract. Disruption of the gut–heart axis, characterized by intestinal hypoperfusion, barrier dysfunction, microbial dysbiosis, and the abnormal production of gut-derived metabolites, plays a pivotal role in sustaining chronic inflammation, metabolic dysfunction, and adverse cardiovascular remodeling. Accumulating evidence supports the concept that the gut microbiota actively contributes to HF progression and prognosis, rather than representing a passive consequence of advanced disease.

Within this framework, next-generation heart failure therapies such as SGLT2 inhibitors and sacubitril/valsartan represent more than purely cardio-centric treatments. Beyond their well-established hemodynamic and neurohormonal benefits, these agents seem to exert pleiotropic effects on endothelial function, systemic inflammation, metabolic pathways, and tissue perfusion—mechanisms that are closely linked to intestinal homeostasis.

Preclinical studies and emerging data suggest that both SGLT2 inhibitors and ARNI therapy may have particular actions on the gut microbiota, favoring bacterial taxa associated with improved barrier integrity and beneficial metabolite production while reducing pro-inflammatory and dysmetabolic microbial signatures.

Despite these promising observations, several important limitations still affect this field. The first is the absence of robust and consistent clinical trials. In fact, the majority of the evidence comes from preclinical studies conducted on rats in settings that often do not involve heart failure. The only human data from trials with SGLT2 inhibitors are limited and need to be expanded to ensure credibility. There is also significant heterogeneity between existing databases, mainly because there is no appropriate, specific scale to be used when comparing the taxonomy and the functions associated with the human microbiome. The latter, together with the absence of a quantitative definition of microbial dysbiosis, represents an important limitation. Another notable limitation is the considerable heterogeneity of HF populations, which influences the microbiota composition, as well as variations in the use of other medications or simply patients’ age. Most studies do not adjust the outcomes according to these factors, and this makes it difficult to distinguish possible drug effects from indirect or exogenous sources.

Future research should focus on well-designed longitudinal clinical human studies integrating metagenomics, metabolomics, and immunophenotyping to clarify the mechanistic links between novel HF therapies and the gut microbiome. The identification of microbiota-derived biomarkers may improve risk stratification, help in predicting treatment responses, and guide therapeutic decision-making. Furthermore, combining pharmacological therapy with targeted dietary interventions, prebiotics, probiotics, or other microbiota-modulating strategies may represent a novel adjunctive approach to enhance treatment efficacy and improve patient outcomes.

In conclusion, the intersection between heart failure pharmacotherapy and gut microbiota biology represents a rapidly evolving and highly promising field with significant translational potential. A deeper understanding of this complex crosstalk may open up new avenues for the integrated, mechanism-based, and personalized management of heart failure treatment in the years to come.

## Figures and Tables

**Table 1:** Gut microbiota alterations and microbiota-derived metabolites involved in heart failure pathophysiology.

Alteration/Metabolite	Microbial Origin	Main Mechanisms	Cardiovascular Effects	Clinical/Prognostic Relevance
Gut microbiota dysbiosis	↓ Firmicutes and Bacteroidetes; ↑ Proteobacteria and Actinobacteria	Increased intestinal permeability, bacterial translocation, systemic inflammation	HF progression, myocardial damage, adverse remodeling	Associated with HF severity and worse outcomes [23,24,25,26]
Trimethylamine N-oxide (TMAO)	Hepatic oxidation of gut-derived trimethylamine from choline/carnitine	Oxidative stress, inflammation, mitochondrial dysfunction, sympathetic activation	Myocardial hypertrophy and fibrosis, endothelial dysfunction, impaired contractility	Strong independent prognostic biomarker in HF [21,23,27,28,29,30,31,32,33]
Phenylacetic acid/phenylacetylglutamine	Microbial phenylalanine metabolism	Metabolic dysregulation, reduced adrenergic responsiveness	Reduced inotropic reserve, increased BNP, maladaptive remodeling	Associated with short-term mortality and adverse prognosis [34,35,36]
Lipopolysaccharide (LPS)	Translocation of Gram-negative bacteria	Immune activation, chronic low-grade inflammation	Myocardial injury, worsening HF	Inversely correlated with cardiac function [23,37]
Short-chain fatty acids (SCFAs)	Fermentation of dietary fibers	Anti-inflammatory effects, metabolic regulation, epigenetic modulation	Cardioprotective effects, improved myocardial function (especially butyrate)	Reduced circulating levels in HF patients [23]
Imidazole propionate (IMP)	Gut-microbial histidine metabolism	Energy metabolism reprogramming, fibrosis, anti-angiogenic effects	LV dysfunction, adverse remodeling, impaired perfusion	Independent predictor of mortality and disease severity [38,39,40]

**Table 2:** Interactions between novel heart failure therapies and the gut microbiota.

Pharmacological Molecule	Evidence Level	Microbiota/Metabolite Changes	Proposed Mechanisms	Potential Clinical Implications
SGLT2 inhibitors (class effect)	Preclinical (non-HF) and exploratory human studies	Modulation of gut microbiota composition; variable changes in Firmicutes/Bacteroidetes ratio; possible increase in SCFA-producing bacteria	Effects on metabolic pathways, systemic inflammation, and intestinal environment	May contribute to cardio-renal and metabolic benefits; findings remain heterogeneous
Dapagliflozin	Preclinical HF and non-HF models; exploratory human HF study	Increased microbial diversity; enrichment of Akkermansia and other taxa; reduced TMAO and increased butyrate (preclinical data)	Coupled vascular and microbiota remodeling; improvement in endothelial function and inflammatory profile	Supports pleiotropic mechanisms beyond glucose lowering; clinical relevance remains to be confirmed [41,44,45,49,50]
Luseogliflozin	Preclinical (non-HF murine metabolic model)	Modulation of amino acid-related microbial pathways; changes in specific taxa (e.g., Syntrophomonadaceae)	Improvement in amino acid metabolism and muscle-related pathways	Potential relevance for metabolic dysfunction and sarcopenic phenotypes; indirect implications for HF [42]
Canagliflozin	Preclinical (T2DM + CVD model)	Partial restoration of Firmicutes/Bacteroidetes balance; reduction in dysbiosis	Reduced inflammation, oxidative stress, and lipid accumulation; improved mitochondrial function	Suggests cardiometabolic benefit possibly mediated by microbiota modulation [43]
Empagliflozin	Exploratory human studies and ongoing clinical trials (HFpEF)	Increased SCFA-producing bacteria; reshaping of microbial composition; longitudinal assessment ongoing	Potential modulation of metabolic and inflammatory pathways; interaction with host–microbiota signaling	May clarify temporal associations between therapy and microbiota changes; causal relationships remain to be established [54]
Sacubitril/valsartan (ARNI)	Preclinical (non-HF and renal/vascular models)	Increased beneficial bacteria taxa (e.g., Lactobacillus, Parabacteroides); reduction in pro-inflammatory signatures	Improvement in intestinal barrier integrity, modulation of RAAS, and reduction in systemic inflammation	Suggests a potential role in gut–heart axis modulation; evidence currently limited to preclinical studies [58,59]
RAAS inhibitors (ACEi/ARB)	Preclinical and exploratory human studies (non-HF populations)	Increased beneficial taxa (e.g., Lactobacillus spp., Odoribacter); reduction in pathogenic bacteria (e.g., Enterobacter, Klebsiella); modulation of SCFAs	Modulation of local intestinal RAAS signaling; improved intestinal perfusion, reduced oxidative stress, and enhanced barrier integrity suggest potential class effect on gut microbiota; relevance in heart failure remains indirect and not specifically established	Potential class effect on gut microbiota; relevance in heart failure remains indirect and not specifically established [61,62,63]

## Data Availability

No new data were created or analyzed in this study. Data sharing is not applicable.
